# Using a Low‐Cost Trackball System to Assess Repeatability of Movement in Small Animals

**DOI:** 10.1002/ece3.72535

**Published:** 2025-12-10

**Authors:** Mikkel Roald‐Arbøl, Elisabeth Yarwood, A. Sofia David Fernandes, Estelle M. Moubarak, Claudia Drees, Jeremy E. Niven, Wiebke Schuett

**Affiliations:** ^1^ School of Life Sciences University of Sussex Brighton UK

**Keywords:** animal personality, automated tracking, carabid, *Carabus problematicus*, ground beetle, locomotion, open‐source software, treadmill

## Abstract

Individual differences in movement patterns are increasingly recognised as important within ecology. In the laboratory, they are, however, often quantified through relatively coarse measures. We describe a low‐cost trackball system incorporating a 3D‐printed holder, commercially available polystyrene ball and implemented with open‐source software. We used this system to record six parameters of walking behaviour (e.g., translational velocity, total rotation) and tested the hypothesis that these walking parameters are repeatable across individuals. We tested 30 ground beetle individuals, *Carabus problematicus*, each in two trackball trials 1 week apart. Individuals were repeatable and differed consistently in several movement parameters (including distance walked, translational velocity and path straightness) but not in others (e.g., total rotation and sinuosity). Trackballs allow quantification of walking parameters for a wide range of animals, enabling identification of individual differences in specific aspects of walking, both fine‐scale and long‐distance aspects of movement. In doing so, trackballs offer novel insights into behavioural ecology, including consistent animal personality differences.

## Graphical abstract

We describe a low‐cost trackball with automated movement capture for use in behavioural ecology. Repeated testing of ground beetles *Carabus problematicus* showed consistent behavioural differences between individuals in movement parameters. Trackballs offer novel insights into behavioural ecology, including consistent animal personality differences.

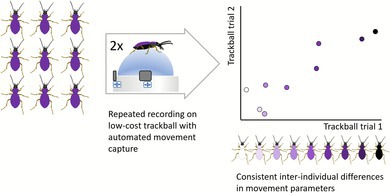

## 1 Introduction

Movement is essential to animal life and impacts multiple levels of organisation (e.g., Wilkinson 2016). Individuals move, for example, to obtain food, locate mates or avoid predators, all influencing individual fitness. These movements also influence population dynamics and distribution, as well as, through species interactions, communities, nutrient flow, and the spread of invasive species or diseases (Bauer and Hoye 2014). Consequently, the study of animal movement is important in many biological disciplines and has received increased attention in recent years (Holyoak et al. 2008; Joo et al. 2022).

Increasingly, researchers recognise the importance of considering individual differences when studying animal movements (e.g., Lubitz et al. 2022; Shaw 2020) and there is growing evidence that conspecifics often differ consistently in movement (e.g., Cote et al. 2010; Harrison et al. 2015). Recent studies have addressed such consistent differences in movement in a personality context (i.e., consistent behavioural differences among individuals, sensu Dall et al. 2004), assessing their ecological and evolutionary consequences (Spiegel et al. 2017). In the laboratory, their characterisation often addresses one aspect, exploration, through novel environment tests in restricted spaces (e.g., Kluen et al. 2012; Montiglio et al. 2010; Schuett et al. 2011) that frequently employ short durations and/or few or coarse metrics such as the number of squares visited or hops made (e.g., David et al. 2011; Dingemanse et al. 2002; Schirmer et al. 2019; Schuett et al. 2018). Other aspects might include running speed in an arena or on a short 2D racetrack (e.g., Norris et al. 2024), time spent active or distance covered in an arena from video recordings (Le Galliard et al. 2013).

Trackballs allow animals to move over large distances providing fine‐scale movement data. Such devices are employed to quantify movement in mechanistic animal behaviour and behavioural neurobiology research, and have been used to study movement in a wide range of animals, including insects (e.g., Clement et al. 2023; Dahmen et al. 2017; Hedwig and Poulet 2005). Previous studies employing trackballs have typically used them to capture metrics that characterise the paths of individuals, such as their orientation, and even quantify the impact of single limb movement on these paths during goal‐directed movements to, for example, auditory or visual stimuli generated by conspecifics (Hedwig and Poulet 2005; Moubarak et al. 2025) or environmental stimuli (Dahmen et al. 2017). Surprisingly, trackball systems have not been used to characterise individual differences in movement in behavioural ecology and allied fields of study. Potential reasons might include unfamiliarity with or lack of knowledge of this method, costs, or technological barriers.

Here, we use a low‐cost trackball system (Moubarak et al. 2025) to characterise multiple aspects of movement and identify consistent individual differences using the ground beetle *Carabus problematicus* as a case study. This trackball system is scalable to different (smaller) species and, we argue, has many potential applications beyond quantifying individual differences including the assessment of fine‐scale movements, the influence of tag weights and the impacts of sensory stimuli. Such applications complement existing techniques, including the use of video recordings, novel environment tests and tracking of animals in the field, opening up new avenues of investigation within behavioural ecology and allied fields.

## 2 Materials and Methods

### 2.1 Animals and Maintenance

Thirty *Carabus problematicus* (11 females, 19 males) (Coleoptera: Carabidae) were collected from woodland surrounding the University of Sussex, East Sussex, UK, from July 2020 to 2021 using live pitfall traps (Yarwood et al. 2021). *Carabus problematicus*, Herbst, 1786 is a flightless, nocturnal ground beetle 24–30 mm in length (Turin et al. 2003). Beetles weighed 0.501 ± 0.08 g (mean ± SD; range: 0.357–0.635 g; *N* = 29) and were maintained under a reversed 12 h:12 h light: dark regime, at 11°C during the light and at 6°C during the dark period in an incubator (Binder, Tuttlingen, Germany). Individuals were housed separately in 10(L) × 7.5(W) × 5(H) cm plastic containers filled with peat and regularly sprayed with water and fed *Tenebrio molitor* pupae *ad libitum*.

### 2.2 Trackball Design

**FIGURE 1:**
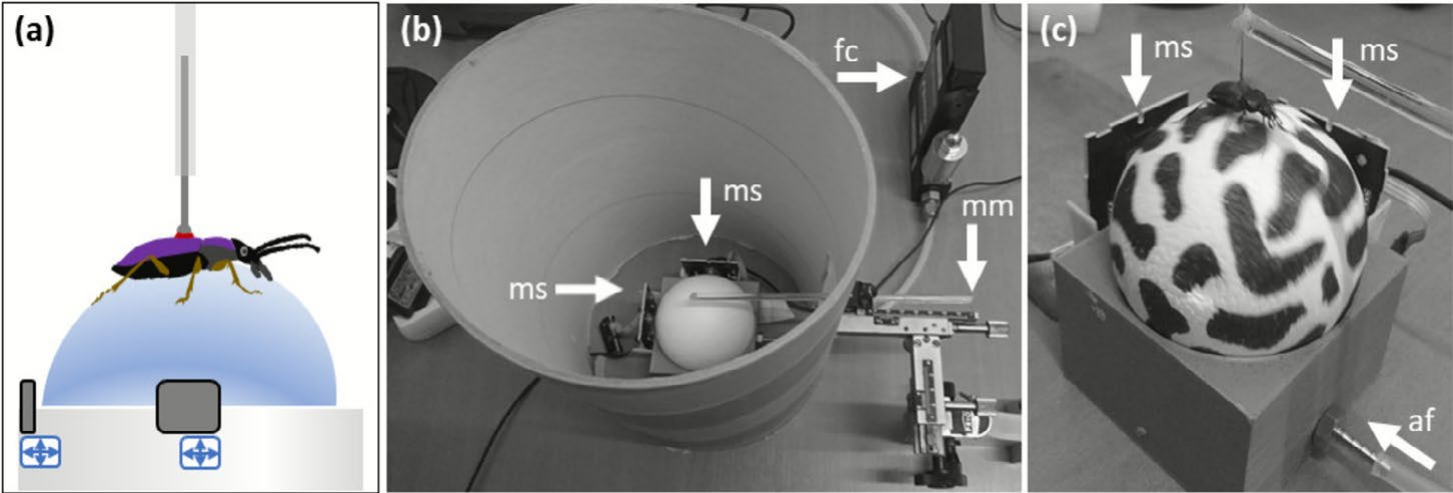
The low‐cost trackball. (a) Schematic of a ground beetle on a trackball. A pin attached to the beetle allows free movement on the trackball. Trackball movements are recorded by mouse sensors (grey squares). (b) Top view on a trackball. Arrows indicate positioning of mouse sensors (ms), mass flow controller (fc) and micromanipulator (mm) with rod used to attach the pin. The white trackball used for illustrative purpose only. (c) Beetle running on trackball lifted by airflow (af).

**FIGURE A1:**
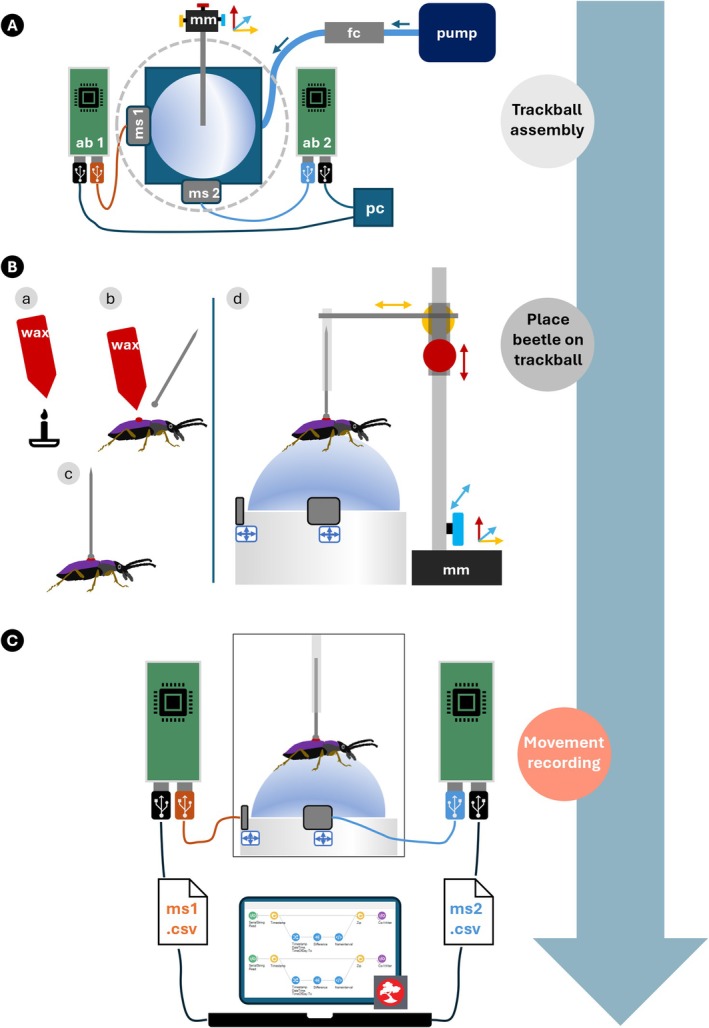
Schematic assembly instructions for setting up and running a trackball system. A pump generates an airflow (arrow) that is regulated by a mass‐flow controller (fc), lifting the polystyrene ball from its cup. Two orthogonally arranged mouse sensors (ms) record trackball movements via Arduino Due microcontroller boards (ab) to the connected computer. The beetle is placed centrally on the trackball using micromanipulator (mm) allowing 3‐dimensional adjustments. Beetles are tethered to an insect pin using a drop of wax attached to the elytra. (A) Top view, (B, C) Side view. (For further details see main text).

**FIGURE A2:**
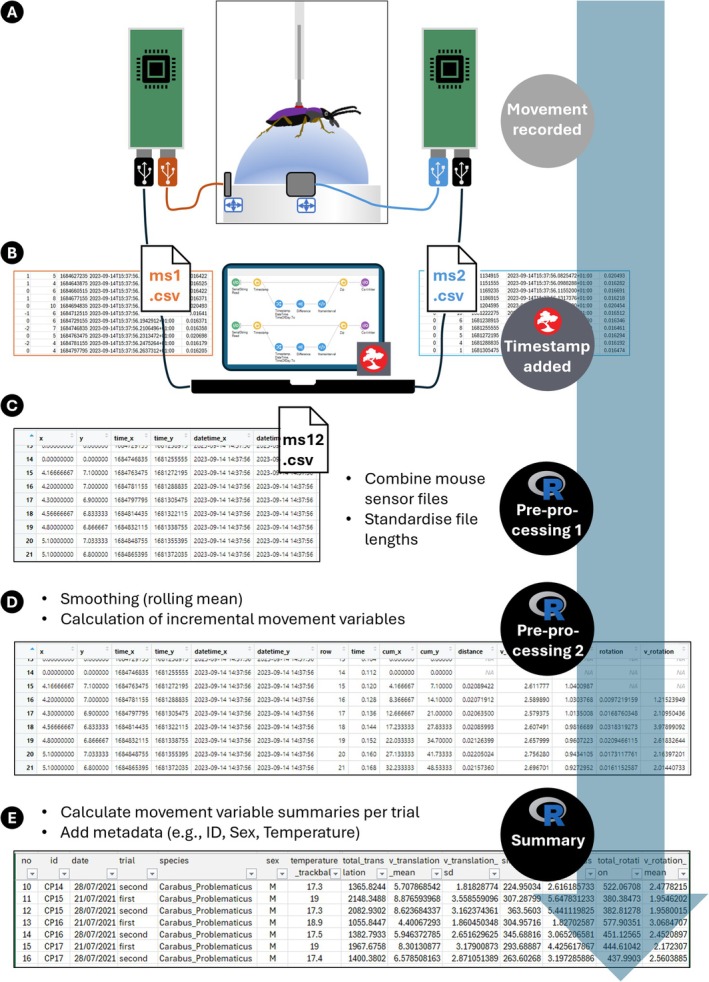
Schematic overview of obtaining and processing trackball data. Bonsai (Lopes et al. 2015) adds time stamps to data from both mouse sensors (A, B). Data are pre‐processed in R, to combine data from both mouse sensors to one .csv file (C, Pre‐processing 1). In a second step movement values are smoothed and incremental movement variables calculated (D). Movement variables are then summarised per trial and information about the trial (metadata) added (E). Pre‐processing steps can be done with R package animovement (version 0.5.1; Roald‐Arbøl 2024).

The polystyrene trackball (8 cm diameter; Skive, Denmark) was held in a custom 3D‐printed (Prusa mk3s, Prusa Research a.s., Prague, Czech Republic; see https://github.com/Sussex‐Neuroscience/NL‐glow‐worm/tree/main/hardware for 3D‐printer file) acrylic half‐sphere cup with a hole (1.6 mm diameter) at the base (Figure 1a–c) connected to an air supply from an ET80 Linear Air Pump (Charles Austen Pumps Ltd., West Byfleet, UK; Figure A1: assembly instructions; the Appendix can be found at the end of this paper). Air pressure was adjusted using a mass flow controller (FMA5523A, OMEGA Engineering, Manchester, UK) to lift the trackball slightly from the cup allowing it to rotate without friction. Optical sensors from two Logitech M500 Wired USB mice (Logitech, Lausanne, Switzerland) were mounted on the cup at 90° to monitor trackball movements in orthogonal (forward‐backward, left–right) planes (Figure 1a–c). The sensors were each connected to a laptop (Dell Latitude 5290, Dell, Texas, USA) via an Arduino Due microcontroller board (Arduino, Massachusetts, USA) running open source *Arduino* Software (*IDE*) version 1.8.15 (Banzi 2009), allowing each sensor to operate in isolation. Bonsai (Lopes et al. 2015) was used to collect the information from both Arduino boards and add time stamps. Bonsai produced one .csv file per mouse sensor that was used for subsequent processing and analyses (Figure A2). The trackball was surrounded by a white cardboard cylinder (~20 cm diameter) (Figure 1b) to eliminate visual stimuli. A summary of equipment needed as well as important aspects, such as specifications, to consider when choosing equipment can be found in Table A1.

### 2.3 Attaching a Tether to the Beetles

Beetles were restrained in modelling clay (Plasticine) and a precision drill (PROXXON GmbH, Föhren, Germany) was used to scratch a small region on the elytral disk to which a vertical metal pin was attached as a tether using red sealing wax (Fred Aldous Ltd., Leeds, UK). The metal pin plus wax weighed 0.03 ± 0.02 g (mean ± SD). Tethered beetles were housed overnight in separate empty maintenance containers prior to testing. Subsequently, individuals were tethered by inserting the pin into a small vertical tube modified from a glass microcapillary (outer diameter 1 mm, inner diameter 0.5 mm) (Harvard Apparatus Ltd., Edenbridge, UK) suspended above the trackball's apex via a horizontal rod attached to a manual micromanipulator (Prior Scientific Instruments, Cambridge, UK) (Figure 1a–c), allowing beetles to adjust their height above the trackball and rotate freely. The rod and micromanipulator were admitted through a small rectangular window removed from the white cylinder (Figure 1b).

### 2.4 Trackball Trials

**FIGURE A3:**
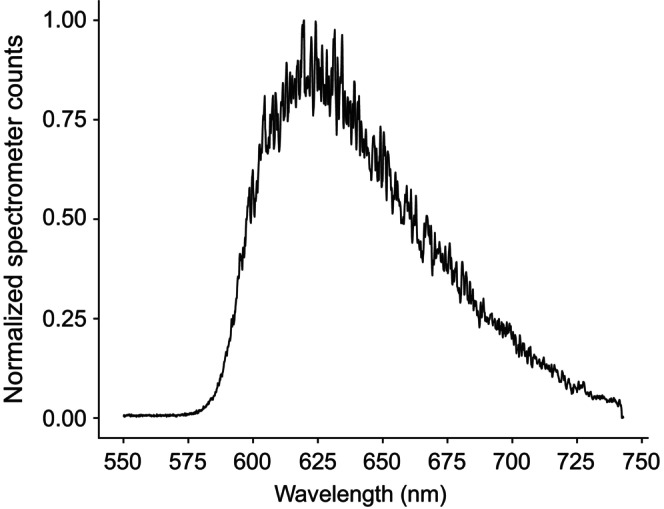
Emission spectrum of the light source used to illuminate the trackball and beetle during trials. The spectrum is dominated by wavelengths in the orange (585–620 nm) and red (625–740 nm). The spectrum was measured with a spectrometer (CCS100/M, Thorlabs, Newton, NJ, USA). We used a commercial light source (Inter IKEA Systems B.V., Leiden, The Netherlands) with an orange/red filter.

Trials were run in July when the beetles are naturally active (Turin et al. 2003). Throughout single 10‐min trials the trackball and beetle were illuminated with red light (Figure A3) to reduce disturbance (Drees et al. 2008). The temperature was recorded continually using a Voltcraft DL‐210TH data logger (Conrad Electronic SE, Hirschau, Germany) and ranged from 17.1°C to 19.0°C during trackball trials. After each trial the tether and metal pin were removed from the beetle, which was returned to the peat‐filled container. All beetles had a second trial 7 days after the first.

### 2.5 Trackball Data Processing

The files we obtained from Bonsai (.csv) for each mouse sensor on each trial contained timestamps and ~60 x and y coordinates per second in dots per inch (dpi). Data were processed in R (version 4.3.3; R Core Team 2024) using R scripts that were later combined into the animovement package (version 0.5.1; Roald‐Arbøl 2024). Files were trimmed to the minimum number of measurements across all trials and combined into a single file per trial. A rolling mean (30 measurements centred on each measurement) was used to smooth x and y data (i.e., *N* = 30,277 measures per file). Subsequently, six movement variables (a‐f) were calculated. (a) For each time increment the distance moved was calculated using the Pythagorean theorem and a calibration (394 dots per cm; Appendix A1: Calibration of trackball) applied to convert into cm. The total distance (cm) was the sum of these incremental distances. (b) The mean translational velocity (cm/s) was the mean of the incremental distances (non‐movements excluded) multiplied by the sampling rate (~60 Hz). (c) From all simultaneous movements to x and y between two frames, we calculated the total rotation (rad). Specifically, from the arctangent of the specific x and y coordinates we obtained the direction. The difference between the absolute direction of time *n* − 1 and the absolute direction of time *n* gave the rotation at each time increment. Assuming that beetles could only rotate through a small angle to reach the current direction within 1/60s, for rotations > π, we calculated 2 × π‐rotation. If both absolute directions were identical, rotation was zero. The sum of all rotations was the total rotation. (d) The mean rotational velocity (rad/s) was calculated across all incremental rotations multiplied by the sampling rate. (e) Sinuosity (dimensionless, 0–1) was the straight‐line distance between the start and end point divided by the total distance moved (e.g., Benhamou 2004). (f) Path straightness (cm/rad) was the total distance divided by the total rotation.

### 2.6 Statistical Analysis

Statistical analyses were run in R. Associations between trackball variables (total distance, total rotation, translational velocity, rotational velocity, sinuosity, path straightness) were tested using Spearman rank correlations separately for each trial series. These analyses allowed us to test for links between trackball variables across individuals and to identify redundant variables. We assessed whether trackball variables were repeatable across individuals (i.e., whether individuals differed consistently from one another and showed personality differences in movement) with linear mixed models with Gaussian error structure (LMMs) and beetle ID as random intercept, using the rptR package (version 0.9.22, Stoffel et al. 2017). The repeatability is the amount of total variation (sum of among‐individual and within‐individual variation) that is explained by among‐individual variation (Lessells and Boag 1987), with a value approaching 1 indicating that individuals are highly consistent in their behaviour but greatly differ from one another. If needed to conform with LMM assumptions, as assessed through model diagnostic plots, the response variables were transformed. Because transformation of response variables did not improve diagnostic plots in all cases, we also assessed consistent individual differences in movement variables with Spearman rank correlations and their 95% confidence intervals (CI) (RVAideMemoire package version 0.9‐83‐7; Hervé 2023). All 95% CIs were based on 1000 bootstrap iterations and repeatability values/correlation coefficients were interpreted as being significant if CIs did not include zero.

### 2.7 Ethics Statement

The methods were carried out in accordance with the guidelines of the Association for the Study of Animal Behaviour and approved by the Animal Welfare and Ethical Review Body of the University of Sussex.

## 3 Results

**FIGURE 2:**
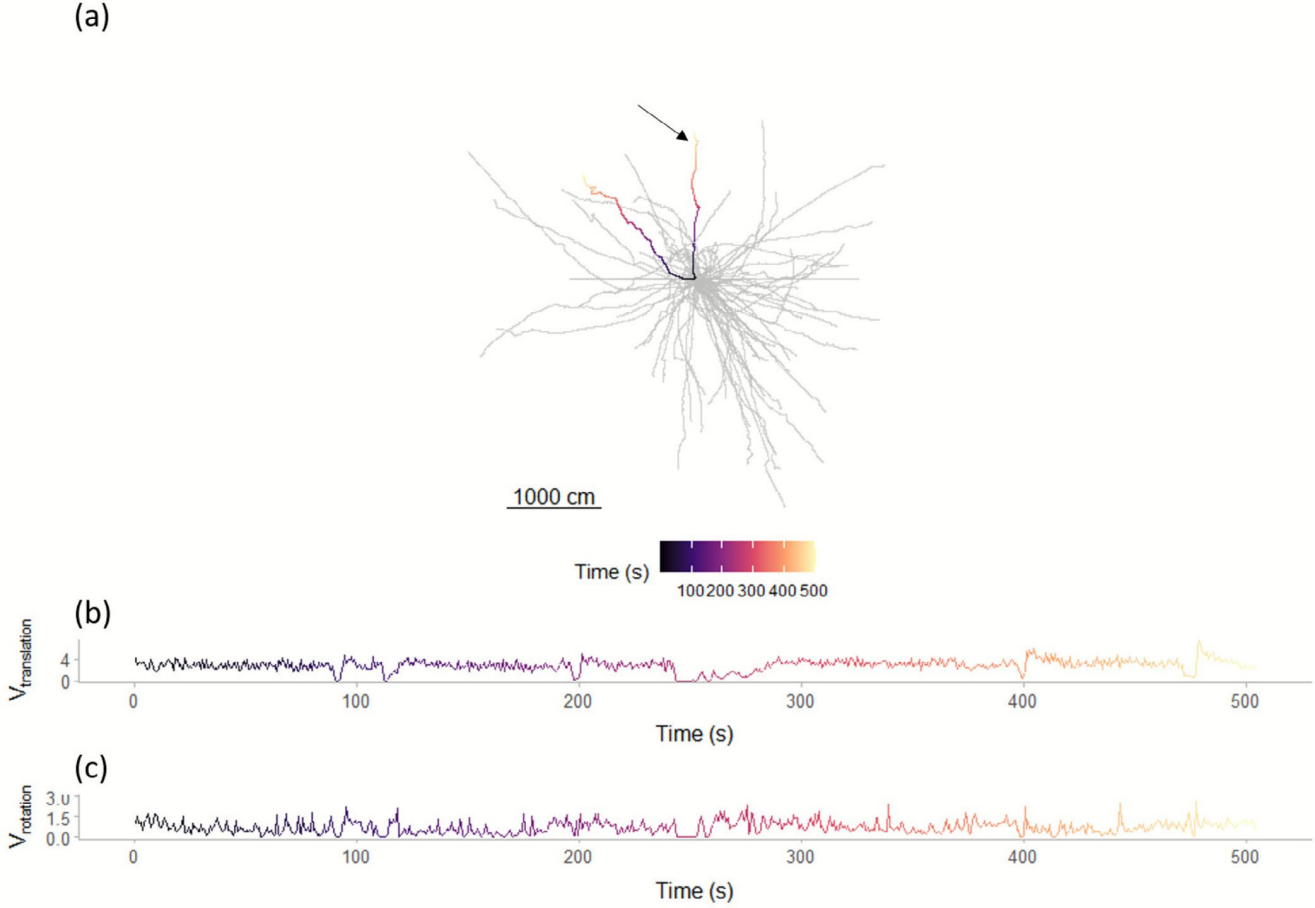
Separation of translational and rotational components of paths walked by beetles on a trackball. (a) Paths walked by 30 individual beetles (grey and coloured lines). Two trials are shown for each beetle. For one individual, these are coloured (starting from black). (b) The velocity of translation (*V*_translation_) during a 500 s period of a single path [marked with arrow in (a)]. (c) As in (b) but for the velocity of rotation (*V*_rotation_).

*Carabus problematicus* individuals on the trackball walked in various directions throughout the trials (Figure 2a). Their paths consisted of translational and rotational components (Figure 2b,c).

**FIGURE 3:**
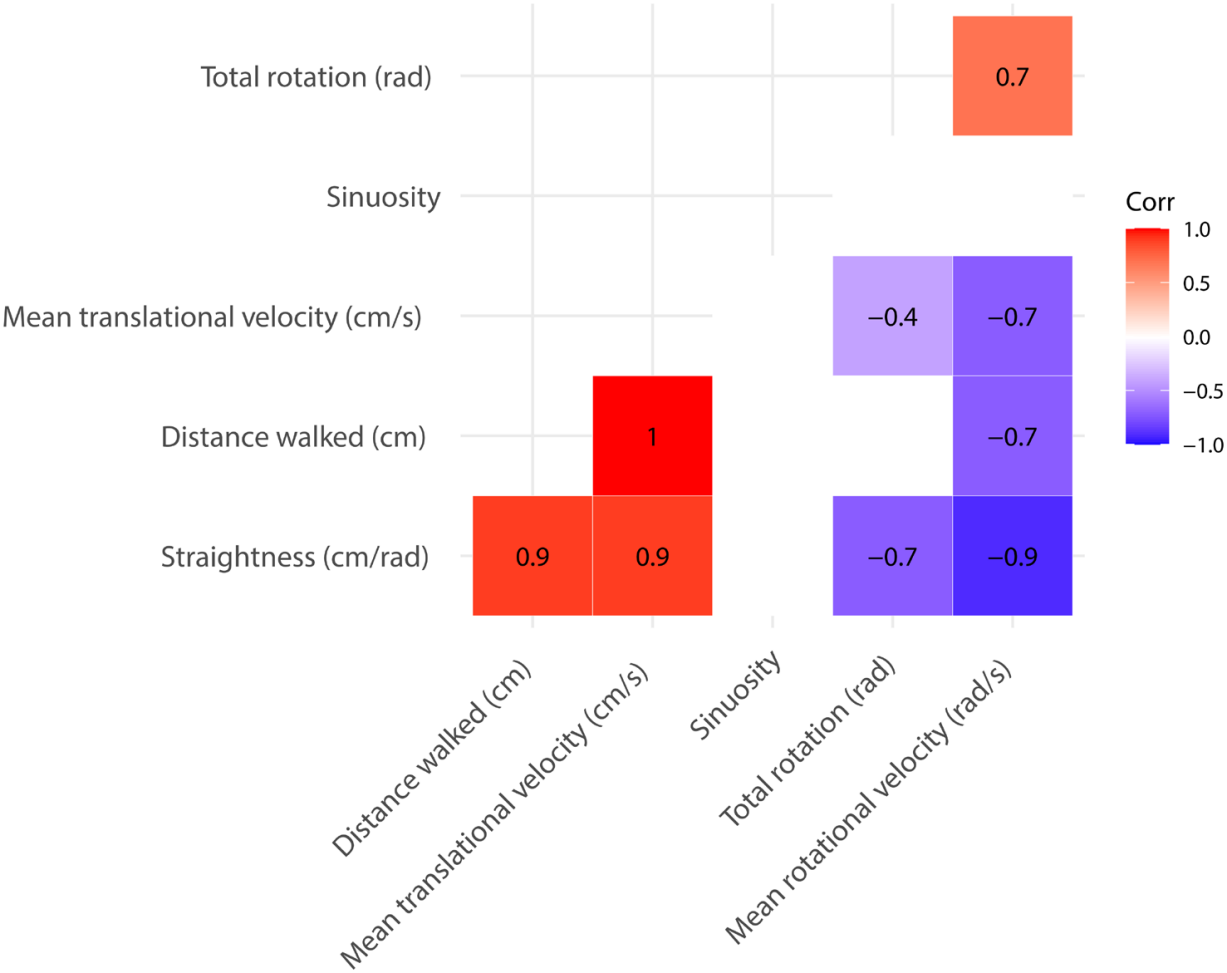
Several variables measured from the trackball were correlated across individual *Carabus problematicus* beetles. Spearman rank coefficients are shown for significant correlations (*p* < 0.05). Data include the first trackball trial per individual (*N* = 30).

**FIGURE A4:**
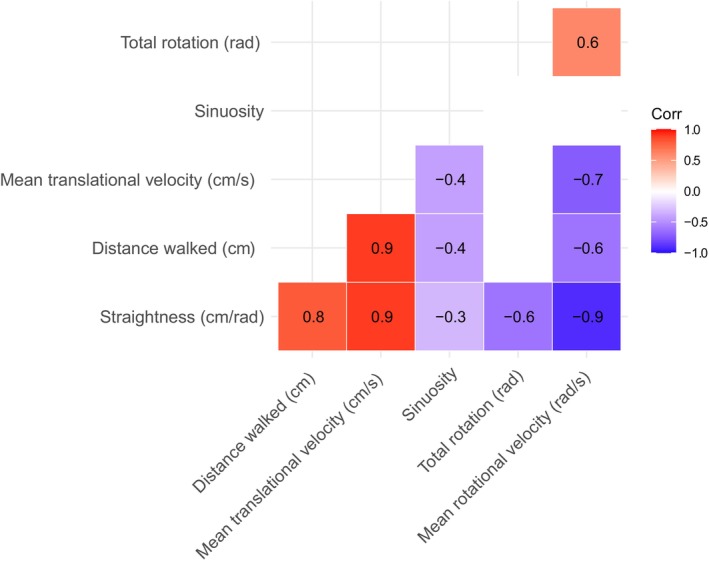
Correlations between trackball variables across individual *Carabus problematicus* beetles. Shown are Spearman rank coefficients for significant correlations (*p* < 0.05); data include the second trackball trial per individual (*N* = 30).

Most trackball variables were significantly correlated across individuals (Figure 3) but only path straightness was highly correlated (*R*_s_ ≥ 0.9) with several other variables and hence combined similar aspects of beetle movement in one variable. Correlations were similar whether considering data from the first or second trial (Figure 3 and Figure A4).

**FIGURE 4:**
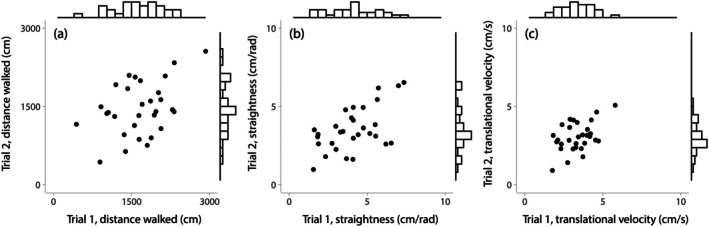
Movement variables from the trackball on two separate trials per individual were positively correlated across individuals (*N* = 30 individuals): (a) Distance walked, (b) path straightness and (c) translational velocity. The histograms show among‐individual variation in these variables in each of the two trials.

Beetles greatly differed in their movements from one another and for some variables, these differences were consistent across time, that is, individuals showed consistent movement differences. For example, beetles walked between 441 and 2924 cm in their first 10‐min trial on the trackball (mean ± SD: 1657 ± 524 cm) and covered similar distances in the second trials (436–2558 cm; mean ± SD: 1460 ± 508 cm; Figure 4a). The distance walked was positively correlated and repeatable between trials (Table 1 and Figure 4a). Similarly, translational velocity and path straightness were positively correlated and repeatable between trials (Table 1 and Figure 4b,c). Conversely, total rotation and rotational velocity were neither correlated nor repeatable, and sinuosity was not correlated, between trials (Table 1).

**TABLE 1:** Repeatabilities (*r*) and Spearman rank correlations (*R*_s_) for trackball variables. Bold test statistics denote significance. *N* = 60 trials of 30 individuals.

Response	Method	*r* or *R*_s_ [95% CI]	Transformation
Total distance	Repeatability	**0.35 [0.02; 0.62]**	sqrt
	Spearman	**0.37 [0.03; 0.64]**	
Total rotation	Repeatability	0 [0.00; 0.34]	
	Spearman	0.05 [−0.31; 0.40]	
Translational velocity	Repeatability	**0.44 [0.10; 0.68]**	
	Spearman	**0.39 [0.04; 0.67]**	
Rotational velocity	Repeatability	0.31 [0.00; 0.57]	Natural log
	Spearman	0.33 [−0.05; 0.68]	
Sinuosity	Repeatability	**0.42 [0.08; 0.68]**	Arcsine sqrt
	Spearman	−0.21 [−0.59; 0.21]	
Straightness	Repeatability	**0.52 [0.21; 0.74]**	
	Spearman	**0.46 [0.10; 0.73]**	

## 4 Discussion

We demonstrate that low‐cost trackball systems could be used to assess individual differences in or repeatability of locomotion for behavioural ecology and allied disciplines. This could, for instance, be relevant when exploring animal personality differences. Using the ground beetle *Carabus problematicus*, we showed that several movement parameters were repeatable, including a novel measure we derived that combines the total translation and total rotation, termed path straightness. Trackball systems have not to our knowledge been used to assess consistent inter‐individual differences in behaviour previously.

The trackball system we use is broadly comparable to those previously employed to measure the movements of several insect species in other biological disciplines though elements of its construction are less costly. Previous trackball systems have combined aluminium (e.g., Dahmen et al. 2017) or acrylic (e.g., Hedwig and Poulet 2005) holders with machined trackballs. In contrast, our system uses a 3D‐printed cup holder with commercially available polystyrene balls, a combination previously used for bees (e.g., Paulk et al. 2014) and glowworms (Moubarak et al. 2025). The availability of 3D printers and the use of commercially available polystyrene balls reduces trackball construction costs. Moreover, using mouse sensors linked to an Arduino board and recorded by open‐source Bonsai software circumvents the need for an analogue‐to‐digital (A2D) converter coupled with specialised software (e.g., Hedwig and Poulet 2005), further reducing costs and increasing accessibility.

Beetles tethered on our trackball appeared to walk unimpeded over long periods of time and large distances, suggesting that neither trackball nor tether hindered their movement substantially. Data obtained from the trackball is of low dimensionality consisting of the outputs of the two mouse sensors. As such, it is easier to process than video, which often involves large files and laborious tracking of movement (whether manual or automated). While we here only tested individuals for 10 min on the trackball, which was sufficient to identify consistent behavioural differences in movement, the manageable file sizes allow much longer tracking, which may reveal other behaviours, such as random walks (indicating foraging) or resting that can be compared among individuals. Related ground beetles, *Nebria brevicollis* , for example, have successfully been recorded on a trackball for 2 h (M. Roald‐Arbøl 2025). It is also possible that our measures included aspects of escape behaviour, but regardless of the motivation we could show consistent individual differences in several movement variables. Longer trials and/or trials in other settings could now be used to better understand the movement behaviours we observed.

Trackball data is of high resolution and separates translational and rotational components. This allows distinctions to be made between the specific parameters of movement (e.g., translation, rotation) to be tested separately for repeatability. As is the case for *C. problematicus* , only specific parameters of movement may be repeatable and some may be collinear, depending on the species. Primarily it was those parameters linked to translation that were repeatable, whereas those linked to rotation were not. Path straightness was the only variable that correlated highly with several other variables, both those linked to translation and to rotation, and could be used as a single composite movement parameter in our data.

The paths of beetles we tested were largely unidirectional, though no specific direction was favoured. This may be a consequence of surrounding the trackball with an unpatterned white cylinder and illuminating it with only dim red light. Such conditions minimised visual cues, which are appropriate for a nocturnal ground beetle. This contrasts with previous uses of insect trackball systems to study movements or neural activity in response to environmental stimuli (e.g., Dahmen et al. 2017; Hedwig and Poulet 2005; Paulk et al. 2014). The presence of sensory cues may alter the movements of our beetles whilst walking on the trackball. Using our system, however, responses to sensory cues could also be assessed for repeatable, inter‐individual differences in movement.

One difference between beetle movements on the trackball and in other laboratory tests assessing movement aspects in arenas or confined spaces, e.g., exploration in a novel environment (e.g., Schuett et al. 2018), is the absence of barriers on the trackball. The response of beetles to the presence of barriers may be a distinct aspect of movement that is not assessed on the trackball. The trackball, however, does allow distinctions to be drawn between the particular aspects of movement that are repeatable in trials in which animals can cover large distances. Trackballs might be less convenient for use in the field though they have been used there (Dahmen et al. 2017). Finally, we have not explored all possible uses of trackball data here and other movement parameters may be extracted, depending on the question and the biology of the study species. For example, the pauses between bouts of movement can also be quantified as can patterns of interaction between specific aspects of movement (e.g., the alternation of translatory and rotatory movements). Given such considerations, we suggest that trackballs offer a paradigm that provides additional understanding of individual differences and repeatability of movement that is complementary to and can be used alongside classical tests to assess movement.

## Author Contributions

**Mikkel Roald‐Arbøl:** conceptualization (equal), data curation (lead), formal analysis (equal), methodology (equal), resources (equal), software (equal), visualization (equal), writing – review and editing (equal). **Elisabeth Yarwood:** data curation (supporting), investigation (lead), methodology (equal), resources (equal), writing – review and editing (equal). **A. Sofia David Fernandes:** conceptualization (equal), methodology (equal), resources (equal), software (equal), writing – review and editing (equal). **Estelle M. Moubarak:** conceptualization (equal), investigation (supporting), methodology (equal), resources (equal), software (equal), writing – review and editing (equal). **Claudia Drees:** conceptualization (equal), methodology (equal), resources (supporting), supervision (equal), visualization (equal), writing – review and editing (equal). **Jeremy E. Niven:** conceptualization (equal), funding acquisition (equal), methodology (equal), resources (supporting), supervision (equal), writing – original draft (equal), writing – review and editing (equal). **Wiebke Schuett:** conceptualization (equal), formal analysis (equal), funding acquisition (equal), methodology (equal), resources (supporting), supervision (equal), visualization (equal), writing – original draft (equal), writing – review and editing (equal).

## Conflicts of Interest

The authors declare no conflicts of interest.

## Data Availability

The data that support the findings of this study and R code are available on Dryad: https://doi.org/10.5061/dryad.2v6wwq02v (Roald‐Arbøl et al. 2025). Additional information about the trackball hardware and software is available online at https://github.com/Sussex‐Neuroscience/NL‐glow‐worm/tree/main for a smaller version of the trackball.

## Appendix A

**TABLE A1:** A list of key materials required for the construction of the trackball setup described in this manuscript. The table is adapted from https://github.com/Sussex‐Neuroscience/NL‐glow‐worm.

Material	Description	Model	Brand	Comments
**Hardware**				
Trackball	Polystyrene, 8 cm diameter			The sphericity of the trackball should be checked prior to use.
Trackball support (half‐sphere cup)	3D printed (ABS filament)			This requires access to a 3D printer.
Pump	Any with pressure > 10 lpm and constant flow	ET 80 Air Pump	Charles Austen	
Air flow tubes	Any combination that fit the pump, mass flow controller (or regulator) and adaptors/connectors			
Optical mouse (2×)	Corded; Infra‐red light	M500	Logitech	Other brands of optical mouse are available but before purchase the resolution and compatibility should be checked.
Arduino due (2×)			Arduino	
USB to microUSB cables	Any microUSB male to microUSB male			
USB to microUSB connector	Any USB male to microUSB female			
Computer	2 (3) USB ports			Only two USB ports are required for the minimum trackball setup but three would be required if, e.g., a camera was added.
Micromanipulator for tethering	Any that fits the end of the rod and allows fine adjustments			Any equipment that allows for fine adjustment of the position of the insect on the trackball would be feasible. Micromanipulators can be purchased more affordably second‐hand.
Mass flow controller		FMA5523A	Omega Engineering, Manchester, UK	Although a mass flow controller was used for collecting the data presented in this publication, it is not required for the trackball setup; alternatively, a regulator (e.g., Futuris, ~2 to 5 lpm) can be used with a pump. Adding a mass flow controller would be a considerable additional cost.
**Software**				
Arduino IDE		Version 1.8.15	Arduino	Open source
Bonsai				Open source

## Appendix: Calibration of trackball A1

Conversion of the *x* and *y* values obtained by the mouse sensor was done by calibrating each recorded displacement point with the theoretical spatial resolution of the mouse sensor (reported in the datasheet as 394 dots per cm). Additionally, a manual control of this calibration factor was conducted to confirm the accuracy of the theoretical spatial resolution. To do so, the polystyrene ball was marked with a 10 cm line divided in 1 cm increments and manually moved in front of the sensor in four directions (towards the sensor, backwards, and to the left and right of the sensor) along the 10 cm of the markings. The recorded displacements were then calibrated using the theoretical spatial resolution of the mouse sensor and compared with the theoretical distance moved (10 cm). The calibrated path measured on average a 10.2 ± 1.7 cm (mean ± SD; *n* = 4) displacement; confirming the accuracy of the spatial resolution used for distance calibration.

## References

[ece372535-bib-0001] Banzi, M. 2009. Getting Started With Arduino. O'Reilly Media, Inc.

[ece372535-bib-0002] Bauer, S. , and B. J. Hoye . 2014. “Migratory Animals Couple Biodiversity and Ecosystem Functioning Worldwide.” Science 344: 1242552. 24700862 10.1126/science.1242552

[ece372535-bib-0003] Benhamou, S. 2004. “How to Reliably Estimate the Tortuosity of an Animal's Path: Straightness, Sinuosity, or Fractal Dimension?” Journal of Theoretical Biology 229: 209–220. 15207476 10.1016/j.jtbi.2004.03.016

[ece372535-bib-0004] Clement, L. , S. Schwarz , and A. Wystrach . 2023. “An Intrinsic Oscillator Underlies Visual Navigation in Ants.” Current Biology 33: 411–422. 36538930 10.1016/j.cub.2022.11.059

[ece372535-bib-0005] Cote, J. , J. Clobert , A. Brodin , S. Fogarty , and A. Sih . 2010. “Personality‐Dependent Dispersal: Characterization, Ontogeny and Consequences for Spatially Structured Populations.” Philosophical Transactions of the Royal Society, B: Biological Sciences 365: 4065–4076. 10.1098/rstb.2010.0176 PMC2992741 21078658

[ece372535-bib-0006] Dahmen, H. , V. L. Wahl , S. E. Pfeffer , H. A. Mallot , and M. Wittlinger . 2017. “Naturalistic Path Integration of *Cataglyphis* Desert Ants on an Air‐Cushioned Lightweight Spherical Treadmill.” Journal of Experimental Biology 220: 634–644. 28202651 10.1242/jeb.148213

[ece372535-bib-0007] Dall, S. R. X. , A. I. Houston , and J. M. Mcamara . 2004. “The Behavioural Ecology of Personality: Consistent Individual Differences From an Adaptive Perspective.” Ecology Letters 7: 734–739.

[ece372535-bib-0008] David, M. , Y. Auclair , and F. Cézilly . 2011. “Personality Predicts Social Dominance in Female Zebra Finches, *Taeniopygia guttata* , in a Feeding Context.” Animal Behaviour 81: 219–224.

[ece372535-bib-0009] Dingemanse, N. J. , C. Both , P. J. Drent , K. Van Oers , and A. J. Van Noordwijk . 2002. “Repeatability and Heritability of Exploratory Behaviour in Great Tits From the Wild.” Animal Behaviour 64: 929–938.

[ece372535-bib-0010] Drees, C. , A. Matern , and T. Assmann . 2008. “Behavioural Patterns of Nocturnal Carabid Beetles Determined by Direct Observations Under Red‐Light Conditions.” In Back to the Roots and Back to the Future? Towards a New Synthesis Between Taxonomic, Ecological and Biogeographical Approaches in Carabidology, edited by L. Penev , T. Erwin , and T. Assmann . Pensoft Publisher.

[ece372535-bib-0011] Harrison, P. M. , L. F. G. Gutowsky , E. G. Martins , D. A. Patterson , S. J. Cooke , and M. Power . 2015. “Personality‐Dependent Spatial Ecology Occurs Independently from Dispersal in Wild Burbot (*Lota lota*).” Behavioral Ecololgy 26: 483–492.

[ece372535-bib-0012] Hedwig, B. , and J. E. A. Poulet . 2005. “Mechanisms Underlying Phonotactic Steering in the Cricket *Gryllus bimaculatus* Revealed With a Fast Trackball System.” Journal of Experimental Biology 208: 915–927. 15755890 10.1242/jeb.01452

[ece372535-bib-0013] Hervé, M. 2023. “Aide‐Mémoire de Statistique Appliquée à la Biologie—Construire Son Etude et Analyser les Résultats à L'aide du Logiciel R.”

[ece372535-bib-0014] Holyoak, M. , R. Casagrandi , R. Nathan , E. Revilla , and O. Spiegel . 2008. “Trends and Missing Parts in the Study of Movement Ecology.” Proceedings of the National Academy of Sciences 105: 19060–19065. 10.1073/pnas.0800483105 PMC2614715 19060194

[ece372535-bib-0015] Joo, R. , S. Picardi , M. E. Boone , et al. 2022. “Recent Trends in Movement Ecology of Animals and Human Mobility.” Movement Ecology 10: 26. 35614458 10.1186/s40462-022-00322-9 PMC9134608

[ece372535-bib-0016] Kluen, E. , S. Kuhn , B. Kempenaers , and J. E. Brommer . 2012. “A Simple Cage Test Captures Intrinsic Differences in Aspects of Personality Across Individuals in a Passerine Bird.” Animal Behaviour 84: 279–287.

[ece372535-bib-0017] Le Galliard, J.‐F. , M. Paquet , M. Cisel , and L. Montes‐Poloni . 2013. “Personality and the Pace‐Of‐Life Syndrome: Variation and Selection on Exploration, Metabolism and Locomotor Performances.” Functional Ecology 27: 136–144.

[ece372535-bib-0018] Lessells, C. M. , and P. T. Boag . 1987. “Unrepeatable Repeatabilities—A Common Mistake.” Auk 104: 116–121.

[ece372535-bib-0019] Lopes, G. , N. Bonacchi , J. Frazão , et al. 2015. “Bonsai: An Event‐Based Framework for Processing and Controlling Data Streams.” Frontiers in Neuroinformatics 9: 7. 25904861 10.3389/fninf.2015.00007 PMC4389726

[ece372535-bib-0020] Lubitz, N. , M. Bradley , M. Sheaves , N. Hammerschlag , R. Daly , and A. Barnett . 2022. “The Role of Context in Elucidating Drivers of Animal Movement.” Ecology and Evolution 12: e9128. 35898421 10.1002/ece3.9128 PMC9309038

[ece372535-bib-0021] Montiglio, P.‐O. , D. Garant , D. Thomas , and D. Réale . 2010. “Individual Variation in Temporal Activity Patterns in Open‐Field Tests.” Animal Behaviour 80: 905–912.

[ece372535-bib-0022] Moubarak, E. M. , A. S. David Fernandes , A. J. Stewart , and J. E. Niven . 2025. “Multiple Effects of Artificial Lighting at Night on Male Glow‐Worms' Mate Searching Behaviour.” bioRxiv, 2025.02. 18.638807.

[ece372535-bib-0023] Norris, M. C. , T. R. Robbins , and D. A. Warner . 2024. “Locomotor Performance Differs Between Foraging and Predator Escape but is not Related to Survival in Hatchling Lizards.” Biological Journal of the Linnean Society 141: 102–117.

[ece372535-bib-0024] Paulk, A. C. , J. A. Stacey , T. W. J. Pearson , et al. 2014. “Selective Attention in the Honeybee Optic Lobes Precedes Behavioral Choices.” Proceedings of the National Academy of Sciences 111: 5006–5011. 10.1073/pnas.1323297111 PMC3977245 24639490

[ece372535-bib-0026] R Core Team 2024. R: A Language and Environment for Statistical Computing. R Foundation for Statistical Computing.

[ece372535-bib-0027] Roald‐Arbøl, M. 2024. “Animovement: An R Toolbox for Analysing Animal Movement Across Space and Time.” http://www.roald‐arboel.com/animovement/.

[ece372535-bib-0028] Roald‐Arbøl, M. 2025. “Exploring Animal Behaviour Across Timescales, From Movement to Metabolism.” PhD Thesis. University of Sussex.

[ece372535-bib-0040] Roald‐Arbøl, M. , E. Yarwood , A. S. David Fernandes , et al. 2025. “Using a Low‐cost Trackball System to Assess Repeatability of Movement in Small Animals: Data & R code.” [Dataset]. Dryad. 10.5061/dryad.2v6wwq02v. PMC12690218 41383204

[ece372535-bib-0029] Schirmer, A. , A. Herde , J. A. Eccard , and M. Dammhahn . 2019. “Individuals in Space: Personality‐Dependent Space Use, Movement and Microhabitat Use Facilitate Individual Spatial Niche Specialization.” Oecologia 189: 647–660. 30826867 10.1007/s00442-019-04365-5 PMC6418052

[ece372535-bib-0030] Schuett, W. , S. R. X. Dall , and N. J. Royle . 2011. “Pairs of Zebra Finches With Similar ‘personalities’ Make Better Parents.” Animal Behaviour 81: 609–618.

[ece372535-bib-0031] Schuett, W. , B. Delfs , R. Haller , et al. 2018. “Ground Beetles in City Forests: Does Urbanization Predict a Personality Trait?” PeerJ 6: e4360. 29479494 10.7717/peerj.4360 PMC5824674

[ece372535-bib-0032] Shaw, A. K. 2020. “Causes and Consequences of Individual Variation in Animal Movement.” Movement Ecology 8: 12. 32099656 10.1186/s40462-020-0197-x PMC7027015

[ece372535-bib-0033] Spiegel, O. , S. T. Leu , C. M. Bull , and A. Sih . 2017. “What's Your Move? Movement as a Link Between Personality and Spatial Dynamics in Animal Populations.” Ecology Letters 20: 3–18. 28000433 10.1111/ele.12708

[ece372535-bib-0034] Stoffel, M. A. , S. Nakagawa , and H. Schielzeth . 2017. “rptR: Repeatability Estimation and Variance Decomposition by Generalized Linear Mixed‐Effects Models.” Methods in Ecology and Evolution 8: 1639–1644.

[ece372535-bib-0035] Turin, H. , L. Penev , and A. Casale . 2003. The Genus Carabus in Europe. Pensoft Publishers, Sofia.

[ece372535-bib-0036] Wilkinson M 2016 Restless Creatures: The Story of Life in Ten Movements. Icon Books Ltd.

[ece372535-bib-0037] Yarwood, E. , C. Drees , J. E. Niven , M. Gawel , and W. Schuett . 2021. “Sex Differences in Morphology Across an Expanding Range Edge in the Flightless Ground Beetle, *Carabus hortensis* .” Ecology and Evolution 11: 9949–9957. 34367551 10.1002/ece3.7593 PMC8328432

